# Chorea-Acanthocytosis in a Chinese Family With a Pseudo-Dominant Inheritance Mode

**DOI:** 10.3389/fneur.2018.00594

**Published:** 2018-07-24

**Authors:** Fang Yi, Wenwen Li, Nina Xie, Yafang Zhou, Hongwei Xu, Qiying Sun, Lin Zhou

**Affiliations:** Department of Geriatrics, Xiangya Hospital, Central South University, Changsha, China

**Keywords:** chorea-acanthocytosis, *VPS13A*, genetic variation, mutation, case report

## Abstract

Chorea-acanthocytosis (ChAc) is a rare neurodegenerative movement disorder with variable clinical features, including movement disorders, cognitive decline, myopathy, neuropathy, behavioral changes, seizures and acanthocytosis. The majority of ChAc patients display an autosomal recessive mode of inheritance. A pseudodominant way of transmission represents only a rare condition. Few studies have reported the clinical status of the obligate carriers of ChAc. Here, we describe a Chinese ChAc family with a novel mutation in the *VPS13A* gene, presenting a pseudo-dominant inheritance mode. Our report further expanded the knowledge of phenotypes of ChAc.

## Introduction

Chorea-acanthocytosis (ChAc) is a rare neurodegenerative movement disorder with variable clinical features, including movement disorders, cognitive decline, myopathy, neuropathy, behavioral changes, seizures and acanthocytosis. The majority of ChAc patients display an autosomal recessive mode of inheritance. A pseudodominant way of transmission represents only a rare condition (1, 2). The main neuropathological feature of ChAc is marked neuronal loss in the caudate and putamen, with sparing of the cortex (3). The causative gene of ChAc is *VPS13A* (vacuolar protein sorting 13A): a large gene located on chromosome 9q21 comprising 73 exons (4). Few studies have reported the clinical status of the obligate carriers of ChAc. Here we reported a Chinese ChAc family and evaluated the clinical features and genetics of the pedigree members.

## Case report

**Figure 1:**
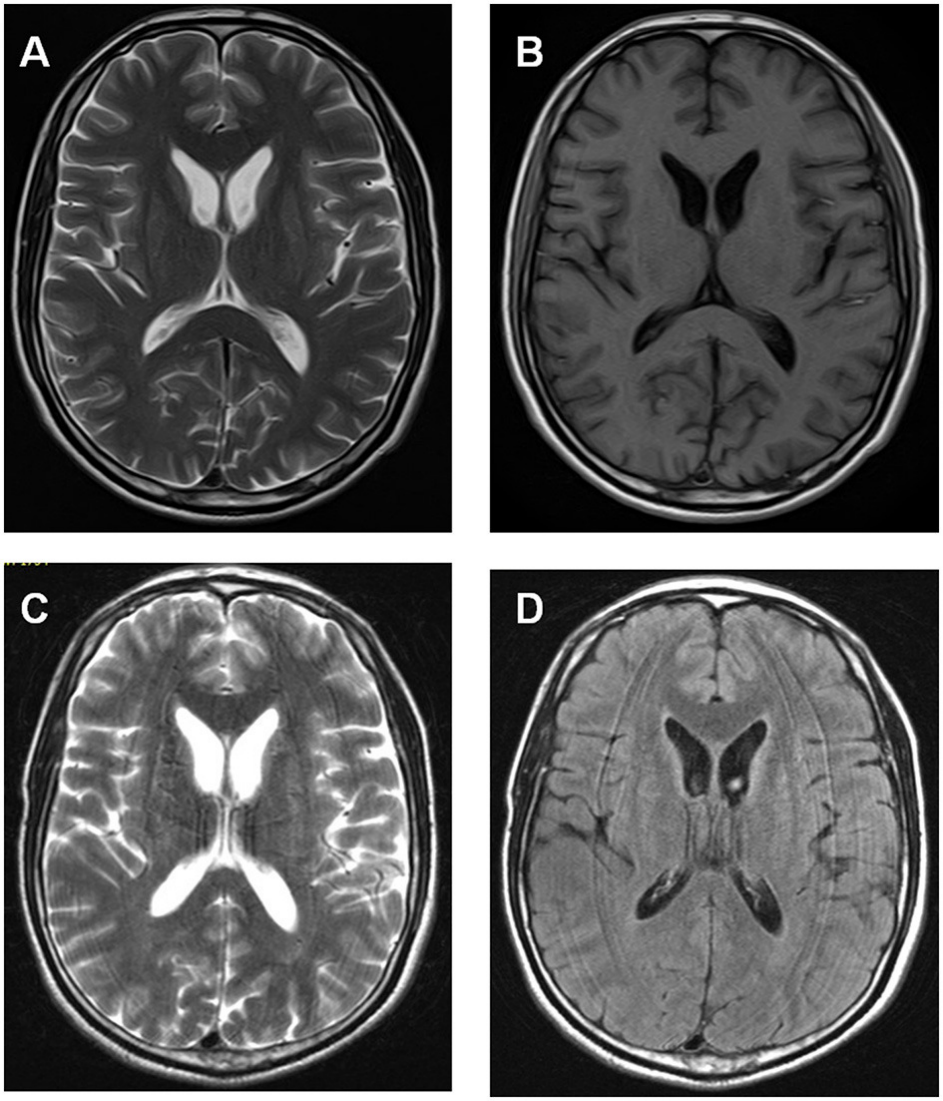
Brain MRI data of the proband showed progressive, symmetrical, mild atrophy of the caudate heads. **(A,B)** Axial T1- and T2-weighted MRI images were taken at 31 year old. **(C,D)** Axial T2-weighted and flare images were taken at 33 year old.

**Figure 2:**
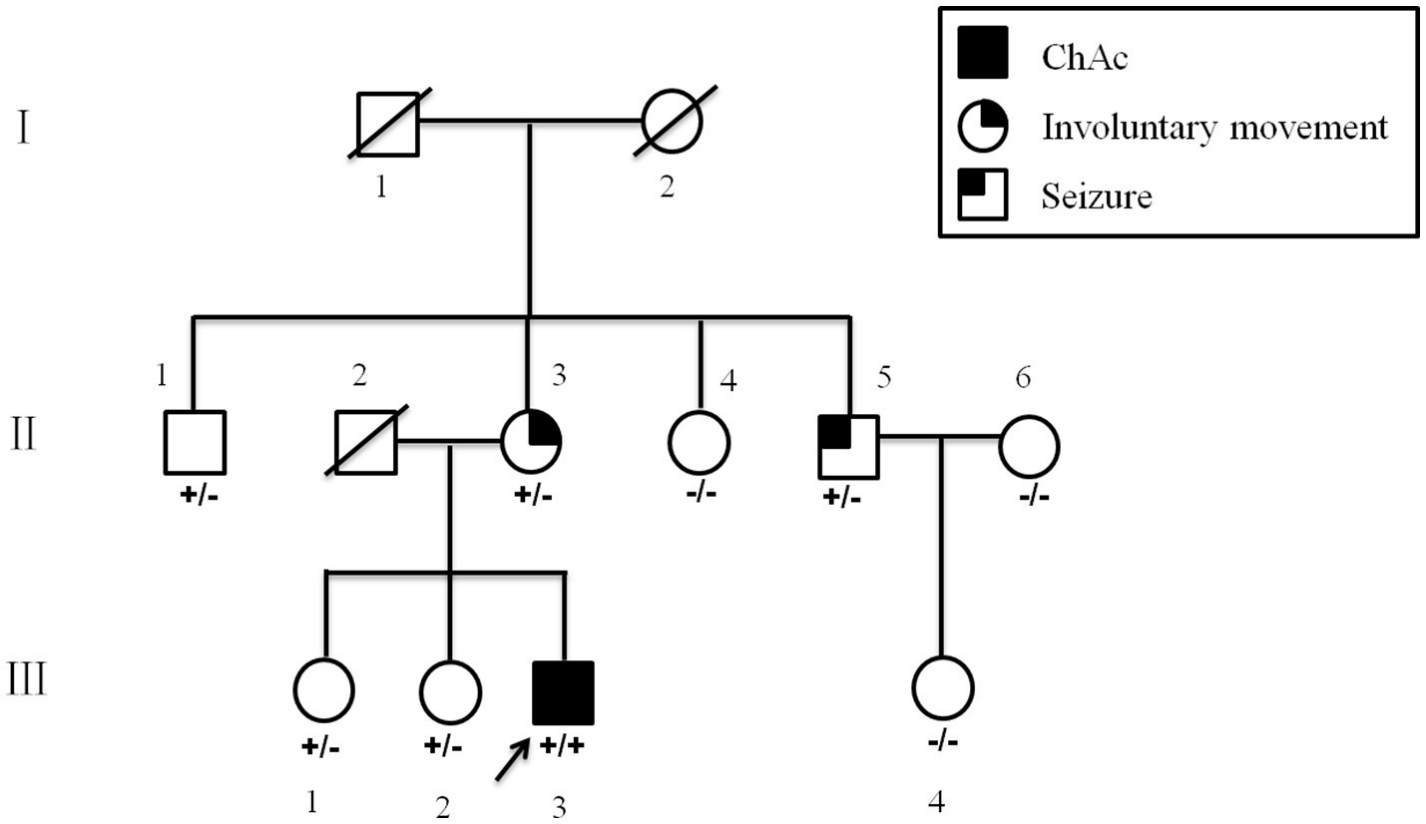
Pedigree of the present family. Blackened symbol indicates individuals with ChAc; blackened symbols at the upper left and upper right indicate involuntary movements and seizures, respectively. The combinations of plus and minus signs indicate the genotypes of *VPS13A*; a plus sign (+) indicates the c.8823C > G mutant allele of *VPS13A*, and a minus sign (−) indicates the normal *VPS13A* allele.

The proband was a 34 year-old right-handed man. From the age of 26 years, involuntary movements of the bilateral lower limbs, associated with dysarthria, grinding teeth and drooling, appeared and gradually worsened. At 31, he suffered from epileptic seizures, which were considered to be generalized tonic-clonic seizure, but antiepileptic drugs had never been administered. One year later, involuntary movements spread to his upper limbs and orofacial automatisms including abnormal tic-like facial movements, tongue protrusion and biting his lips appeared. Then he was treated with haloperidol (2 mg three times a day) and baclofen (10 mg three times a day) for 2 years for his choreic and dystonic problems, but he responded poorly to drug treatments. At age of 34, his involuntary movements gradually spread to his whole body and epileptic seizures increased in frequency. Since the disease onset, the patient had never suffer from psychiatric problems. Neurological examination revealed poor muscle tone and absent deep tendon reflexes in all limbs. Additionally, right positive babinski sign was elicited. Laboratory data revealed elevated creatine kinase level in the peripheral blood. Acanthocytes were found in 4% of cells on the peripheral blood smear test. Doppler ultrasound examination revealed splenomegaly. Brain magnetic resonance imaging (MRI) showed progressive, symmetrical, mild atrophy of the caudate heads (Figure 1). His 24-h continuous electroencephalography (EEG) showed generalized asynchronous theta and epileptiform activity, which mostly originated from the right temporal lobe. A nerve conduction study showed a polyneuropathy, which revealed the right peroneal nerve, right median nerve and bilateral ulnar nerves were partly damaged. His score of Mini Mental Status Examination (MMSE) was 27. The father of the proband did not show any neurological abnormalities and died from pneumonia at 65 years old (Figure 2). The mother of the proband (II-3), a 65-year-old woman, showed mild involuntary movements in her limbs since the age of 45 years (Figure 2). The proband's uncle (II-5), a 52-year-old man, showed mild cognitive impairment (MMSE 24), characterized by memory impairment and had seizures history of 31 years, which were simple partial seizures and treated with antiepileptic drugs (Figure 2). His another uncle (II-1) and two sisters (III-1, III-2) had no neurological clinical symptoms. Brain MRI and peripheral blood smears of the proband's mother, his uncles and two sisters are normal. The clinical picture of the proband was suggestive for ChAc, but the inheritance mode of this family seems to be autosomal dominant.

**Figure 3:**
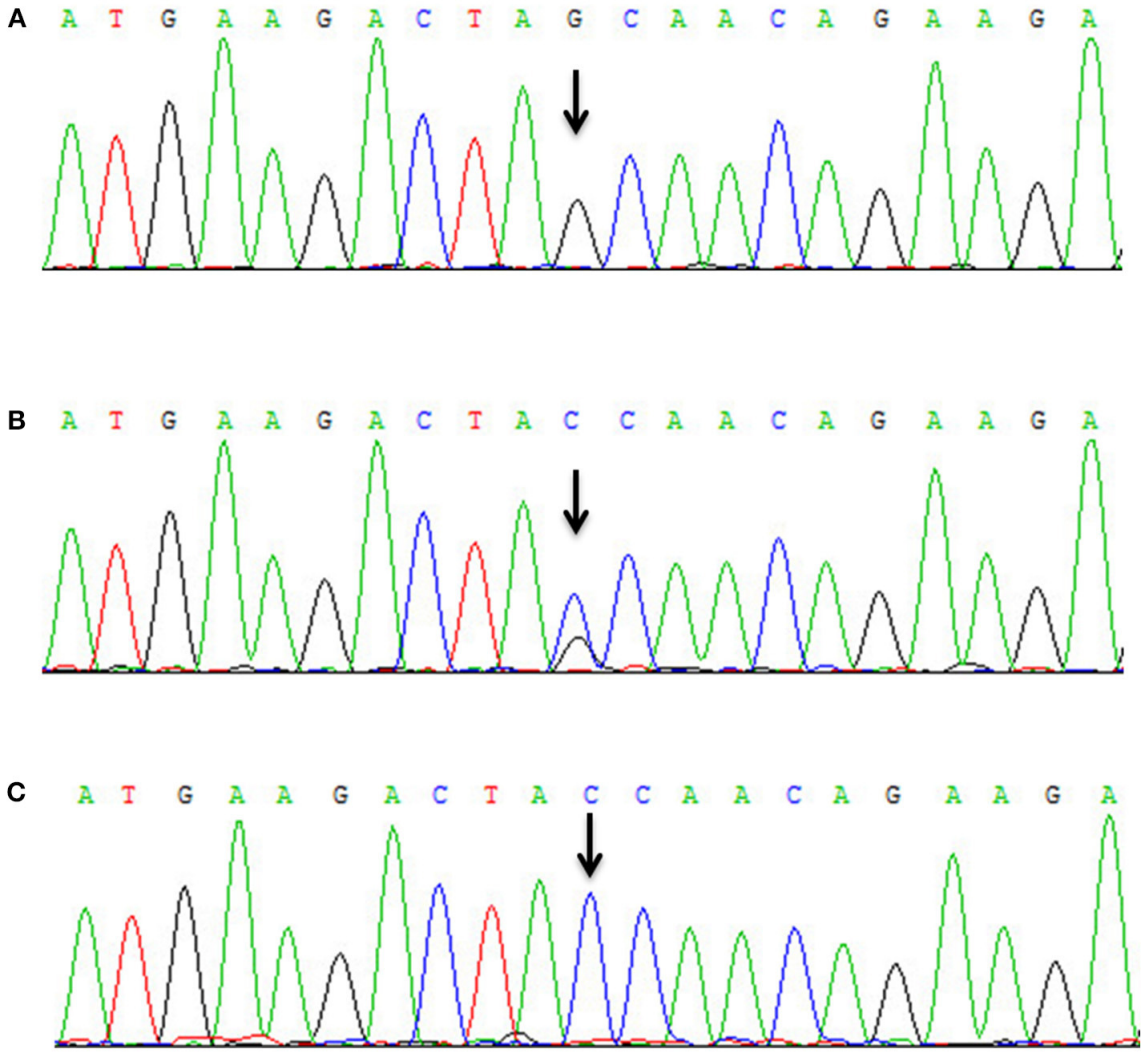
Mutational analysis of *VPS13A* in the ChAc family. **(A)** Sanger sequencing revealed that a homozygous nonsense mutation c.8823C > G (p. Tyr2941^*^) in exon 65 of *VPS13A* was detected in the proband. **(B)** Both II-3 and II-5 had the heterozygous mutation c.8823C > G of *VPS13A*. **(C)** Wild-type sequence.

All patients were of Han nationality from Hunan province, China. Blood specimens and genomic DNA were obtained from family members and 100 control subjects after informed consent. The 73 exons and flanking intronic splice consensus sequences of *VPS13A* were amplified by polymerase chain reaction (PCR) (5, 6). By sequencing, we identified a novel homozygous nonsense mutation c.8823C > G (p. Tyr2941^*^) in exon 65 of *VPS13A* in the proband (Figure 3A). Five members of the family including the proband's mother (II-3) and his uncle (II-5) were detected to be heterozygous for mutation c.8823C > G (Figures 3B,C). The homozygous nonsense mutation c.8823C > G (p. Tyr2941^*^) causes the loss of TPR10 domain of the vacuolar protein sorting 13A protein. This homozygous nonsense mutation c.8823C > G was not detected in 100 healthy controls, thus representing a novel etiology in an ChAc Chinese family. Besides, the mutations in genes responsible for Huntington's disease and McLeod disease were screened and the results were negative.

## Discussion

ChAc is a familial neurodegenerative disorder with various clinical presentations, which may represent a clinical diagnostic challenge, especially when patients' presentation is atypical. A wide range of neuropsychiatric symptoms among patients with ChAc have been reported, which are characterized by frontosubcortical dementia, schizophrenia, paranoia, depression, and marked emotional lability (1, 7). A few studies have reported about atypical phenotypes of ChAc patients (8, 9). The proband in this study showed involuntary movements and seizures, without any neuropsychiatric symptoms, confirming the various clinical presentations of ChAc. Inheritance mode of ChAc typically appears to be autosomal recessive, but autosomal dominant or pseudo-dominant inheritance modes have been reported in a few families (10, 11). In the present study, we found a novel homozygous nonsense mutation c.8823C > G (p. Tyr2941^*^) in exon 65 of *VPS13A* in the proband. 2 out of 5 family members showed signs or symptoms potentially attributable to a heterozygous *VPS13A* mutation. The other three heterozygotes had no neurological clinical symptoms. Thus it is reasonable to assume that the inheritance pattern for this mutation in this family is pseudo-dominant. In addition, there were clinical variabilities in heterozygous mutation carriers: the proband's mother (II-3) showed mild involuntary movements; the proband's uncle (II-5) showed mild cognitive impairment and seizures. This phenomenon indicates that some modifier genes or gene loci, epigenetic factors and environmental conditions might contribute to the pathogenesis of ChAc.

## Conclusion

Our report identified a Chinese ChAc family with a novel mutation in the *VPS13A* gene, presenting a pseudo-dominant inheritance mode. Our report further expanded the knowledge of phenotypes of ChAc.

## Ethics statement

All clinical data in this case report were either provided by the patient and her parents or collected by our team's members with the consent of them. There was no additional invasive test or experimental drugs used out of order for the patient. A written informed consent was obtained from the patient and her parents for the participation in the study and the publication of this report. The case report is exempt from institutional review board approval.

## Author contributions

FY and WL acquired the clinical data, reviewed the literature, and drafted the manuscript. QS and LZ designed the study, oversaw data acquisition, supervised the initial drafting, critically revised the manuscript. HX, NX, and YZ reviewed the literature and revised the manuscript.

### Conflict of interest statement

The authors declare that the research was conducted in the absence of any commercial or financial relationships that could be construed as a potential conflict of interest. The reviewer SP and handling Editor declared their shared affiliation.
